# Unique and overlapping behavioral effects of isoform-specific NRXN1 deletions

**DOI:** 10.1242/dmm.052862

**Published:** 2026-07-23

**Authors:** Amanda E. Friedman, Michele Perni, Josephine Millard, Damjan Karanfilovski, Michael Granato, Philip D. Campbell

**Affiliations:** ^1^ Department of Psychiatry, Perelman School of Medicine, University of Pennsylvania, Philadelphia, PA 19104, USA; ^2^ Department of Genetics, Perelman School of Medicine, University of Pennsylvania, Philadelphia, PA 19104, USA; ^3^ Autism Spectrum Program of Excellence, University of Pennsylvania, Philadelphia, PA 19104, USA; ^4^ Department of Cell and Developmental Biology, Perelman School of Medicine, University of Pennsylvania, Philadelphia, PA 19104, USA

**Keywords:** Neurodevelopment, Behavior, Social, Zebrafish, Psychiatry, Autism

## Abstract

Mutations in the human neurexin 1 (*NRXN1*) gene are associated with neurodevelopmental disorders including autism and schizophrenia. Vertebrate NRXN1 produces three major NRXN1 isoforms, referred to as α, β and γ. Human genetic data suggest that deletions located at the 5′ region of the gene disrupting the α isoform associate mostly with clinical behavioral deficits. Yet, 3′ deletions that disrupt multiple isoforms have also been identified in clinical cases. Whether deletions that selectively affect specific NRXN1 isoforms result in specific behavioral deficits remains unclear. Here, combining larval sensorimotor assays with a custom hands-free social preference assay developed for juvenile zebrafish, we show that zebrafish harboring Nrxn1 deletions that differentially encompass α, β and/or γ isoforms display both unique and overlapping locomotion, sensorimotor behavior, and social behavior deficits. Combined, our results strongly support a model by which domain-selective *nrxn1* deletions predict behavioral phenotypes. Moreover, our results demonstrate compelling relationships between genotype and resulting behavioral phenotypes, providing functional insights into the complexity of NRXN1-associated neuropsychiatric behaviors.

## Teaser

**Summary:** Isoform-specific deletions of Neurexin-1 in zebrafish produce distinct behavioral phenotypes, offering new insights into the complexity of Neurexin-1-associated neurodevelopmental disorders.

## INTRODUCTION

Neurexin 1 (NRXN1) is a pre-synaptic adhesion molecule that plays important roles in synapse formation, maintenance, regulation and function (Südhof, 2017). The *NRXN1* locus is particularly susceptible to non-recurrent copy-number variants (i.e. unique genomic changes that occur at different locations in different individuals), and *NRXN1* deletions have been associated with multiple neurodevelopmental disorders including autism, schizophrenia, intellectual disability and developmental delay (Castronovo et al., 2020). While most individuals ascertained to date have monoallelic deletions, biallelic *NRXN1* deletions have been observed in a small group of individuals diagnosed with Pitt−Hopkins syndrome, who display significant developmental delay (Castronovo et al., 2020; Zweier et al., 2009).

Genomic studies have described three different promoters at human *NRXN1* and mouse *Nrxn1* loci that generate three evolutionarily conserved NRXN1 isoforms known as α, β and γ (Südhof, 2017). While their intracellular domains are identical, their extracellular domains differ, with the α isoform being the longest, the β isoform being intermediate in length and the γ isoform being the shortest. To date, α-isoform-specific exonic deletions located in the 5′ region of *NRXN1* have been strongly associated with clinically defined behavioral phenotypes (Lowther et al., 2017), suggesting that, compared to deletions affecting β or γ isoforms, exonic disruption of the region yielding the NRXN1 α isoform (NRXN1-α) may have a major impact on behavior and, thus, be more pathogenic. However, deletions involving the β isoform have also been identified in individuals with clinical phenotypes (Castronovo et al., 2020; Lowther et al., 2017), suggesting a more nuanced view. Indeed, recent work has suggested that 5′ and 3′ *NRXN1* deletions can act through divergent molecular mechanisms (Flaherty et al., 2019; Fernando et al., 2025) requiring altogether different interventions. Therefore, understanding if and to which degree NRXN1 isoform-specific deletions impact behavioral outcomes is essential to define the spectrum of disease mechanisms. In turn, this knowledge may ultimately predict more fine-tuned and divergent therapeutic interventions.

To date, most behavioral studies of NRXN1 isoforms have focused on mammalian *NRXN1-*α. These studies reveal that in mice, homozygous Nrxn1 deletions specific for NRXN1*-*α cause multiple behavioral abnormalities, including alterations in social behavior, locomotion and anxiety (Grayton et al., 2013; Armstrong et al., 2020; Xu et al., 2023) that are inconsistently seen across studies in heterozygous animals (Grayton et al., 2013; Armstrong et al., 2020; Laarakker et al., 2012; Dachtler et al., 2015). Surprisingly, analogous studies evaluating the function of the NRXN1-β or -γ isoforms and whole-gene *NRXN1* deletions spanning all isoforms have so far not been conducted.

To fill this gap, we used *Danio rerio* (zebrafish), which unlike *Drosophila* and *C. elegans* has maintained a mammalian-like tripartite *nrxn1* promoter architecture. Here, leveraging the strengths of the zebrafish system, we generated zebrafish *nrxn1* whole-gene and isoform-specific deletion lines, and assessed how these deletions affect sensorimotor and social behavior across development. To specifically assess social behavior, we developed a custom hands-free social preference assay for juvenile zebrafish that enables automated stimulus presentation. Our results demonstrate that isoform-specific *nrxn1* deletions resulted in both shared and unique deficits that are both behaviorally and temporally specific. Early in development, we identified opposing sensorimotor phenotypes caused by *nrxn1* deletions specific for α and β isoforms. Later in development, we showed that *nrxn1* is required for social behavior but that deletions specific for Nrxn1*-*α do not fully recapitulate *nrxn1* deletions spanning all isoforms. Together, our data revealed that Nrxn1 deletions that differentially encompass isoforms α, β and γ produce different behavioral outcomes, supporting a more complex and nuanced view of how distinct human *NRXN1* mutations may prompt selective neurodevelopmental phenotypes.

## RESULTS

### *nrxn1a* crispants display robust sensorimotor behavioral phenotypes

The zebrafish genome possesses two *neurexin-1* genes located at two genetically distinct loci, i.e. *nrxn1a* on chromosome 12 and *nrxn1b* on chromosome 13 (Fig. 1A,B)*.* At the protein level, Nrxn1a and Nrxn1b are highly conserved relative to human NRXN1 (Nrxn1a 85% similarity, 76% identity; Nrxn1b 78% similarity, 68% identity) (Hu et al., 2011) and predicted to have identical protein architectures compared to their mammalian counterparts (Fig. 3A,C). To determine the neurodevelopmental function of each gene, we selectively disrupted *nrxn1a* or *nrxn1b* by using a CRISPR-Cas9-based approach that has been previously reported to lead to biallelic null alleles in >90% of animals injected with guide RNA (gRNA) (Kroll et al., 2021). Briefly, three gRNAs that target three non-overlapping sites within *nrxn1a* and *nrxn1b* (Fig. 1A,B) were injected into fertilized wild-type embryos together with Cas9 protein to generate the first generation of mosaic zebrafish lacking *nrxn1a* and *nrxn1b* respectively (i.e. *nrxn1a* and *nrxn1b* crispants). Embryos that were injected with three non-targeting gRNAs and Cas9 were used as controls. *nrxn1a* and *nrxn1b* crispants were viable at 6 days post fertilization (dpf) and did not display obvious gross morphological defects (*nrxn1a*: *n=*117 crispants, *n=*169 controls; *nrxn1b*: *n=*148 crispants, *n=*124 controls; three biological replicates each). We next assessed behavior of *nrxn1a* and *nrxn1b* crispants at 6 dpf, using our previously established and validated pipeline (Campbell et al., 2023; Maroni et al., 2024 preprint) that allows assessment of multiple sensorimotor behaviors. This includes the visual motor response (VMR), whereby larvae are exposed to alternating periods of light and dark with larvae typically increasing movement during dark periods (Emran et al., 2008), responses to brief light or dark flashes (FL or DF, respectively) (Burgess and Granato, 2007b; Wolman et al., 2011), and modulation of the acoustic startle response (ASR) (Wolman et al., 2011; Kimmel et al., 1974; Marsden et al., 2018; Burgess and Granato, 2007a). Using this pipeline, we failed to detect deficits for any of the behaviors assayed in *nrxn1b* crispants (Fig. 1C). We later confirmed these results in *nrxn1b* whole-gene-deletion mutants (*nrxn1b-WD*) (Figs 3C; 4E,J,O; S3E; S4E,J,O). In contrast, *nrxn1a* crispants displayed clear phenotypes that spanned multiple behaviors (Fig. 1C). Specifically, *nrxn1a* crispants displayed reduced movement metrics in response to visual stimuli during the VMR (i.e. distance travelled, average speed and average speed per bout) and the DF (i.e. average distance and duration travelled following a DF) assays, and increased sensitivity to acoustic stimuli in the ASR assay [i.e. percent short latency C-bend in response to loud acoustic stimuli and area under the curve (AUC)] (Fig. 1C). Together, these results suggest that, compared to *nrxn1b*, *nrxn1a* plays more dominant neurodevelopmental roles. Thus, for our isoform-specific analysis, we focused on the *nrxn1a* gene.

**Fig. 1.**
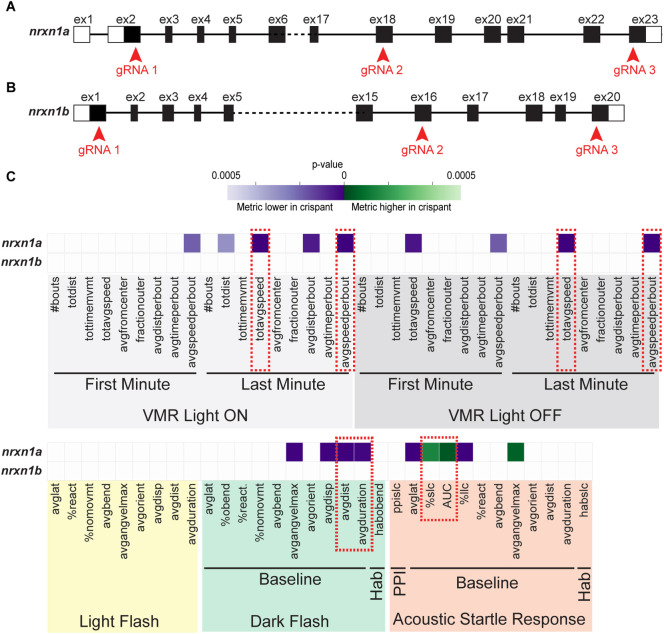
***nrxn1a* crispants display robust sensorimotor behavioral phenotypes.** (A,B) Exon structure of *nrxn1a* (A) and *nrxn1b* (B) showing locations of gRNA targets for crispant generation. (C) Heatmap illustrating *nrxn1a* and *nrxn1b* crispant phenotypes across behavioral metrics. Each row is an individual crispant and all columns represent a behavioral metric. Boxes that are colored designate statistically significant difference in the metric in crispant vs controls based on Student's *t*-test with a Bonferroni-corrected *P*-value of *P*<0.05/94. Visual motor response (VMR) metrics are highlighted in gray, i.e. VMR ‘Light ON’ and VMR ‘Light OFF (light and dark gray background, respectively). Light Flash (LF), Dark Flash (DF) and Acoustic Startle Response (ASR) (yellow, green and orange background colors, respectively). Hab, habituation; PPI, pre-pulse inhibition. Reduced movement metrics during VMR and DF, and increased ASR sensitivity are encircled by dashed red lines. Three independent biological replicates were performed for each gene. *n=*117 crispants, *n=*169 controls (*nrxn1a*); *n=*148 crispants, *n=*124 controls (*nrxn1b*)*.* Data presented are representative of a single biological sample. Behavioral metrics are as follows and have been fully described by Campbell et al. (2023). VMR: Number of bouts (#bouts), Total distance travelled (totdist), Total time moved (tottimemvmt), Average speed (totavgspeed), Average distance from center of the well (avgfromcenter), Fraction of time spent in out rim of well (fractionouter), Average distance per bout (avgdistperbout), Average time per bout (avgtimeperbout), Average speed per bout (avgspeedperbout). DF,LF,ASR: Frequency of reacting (%react), frequency of no movement (%nomovmt), Frequency of O-bend response (%obend), Frequency of short latency C-bend (%slc), Frequency of long latency C-bend (%llc), Average latency to movement (avglat), Average maximum bend angle (avgbend), Average maximum angular velocity (avgangvelmax), Average change in orientation (avgorient), Average displacement (avgdisp), Average distance (avgdist), Average duration (avgduration), Habituation (Hab) of the O-bend response (habobend), Habituation (Hab) of the short latency C-bend (habslc), Pre-pulse inhibition (PPI) of the short latency C-bend (ppislc).

### Zebrafish Nrxn1a α and β isoforms have distinct brain expression patterns

In humans, *NRXN1* encodes three promoter-driven isoforms, termed α, β and γ. Consistent with the tripartite promoter architecture described in mammals, the zebrafish *nrxn1a* locus has retained three major H3K4me3 promoter peaks in ZebrafishENCODE brain data (Fig. S1A) (Bogdanović et al., 2016). While Ensembl (Harrison et al., 2024) currently annotates only transcripts corresponding to the α and β isoforms, a third epigenomic peak supports the presence of a γ-like promoter. Indeed, Ensembl includes an additional RNA-seq-supported *nrxn1a* transcript in 5 dpf larvae originating from a third promoter region, consistent with the γ promoter architecture (Fig. S1B), and RT-PCR of 6 dpf wild-type larvae confirmed the presence of a transcript corresponding to the γ isoform. Together, in accordance with the mammalian literature, this provides compelling evidence for three major promoter-driven Nrxn1a isoforms in zebrafish (Fig. S1B), hereafter referred to as α, β and γ isoforms.

To further validate the existence of the annotated Nrxn1a*-*α and -β isoforms and to define their expression patterns, we performed hybridization chain reaction (HCR) *in situ* with probes specific for α and β isoforms in zebrafish brain at 6 dpf to match our behavioral assessments. Both the Nrxn1a-α and -β isoform transcripts are widely expressed through the brain, and display largely overlapping expression patterns (Fig. 2A). Despite the overall similarities, we detected an enrichment of Nrxn1a-*β* mRNA signal in an anterior region of the forebrain bilaterally (Fig. 2A,A′), corresponding to the pallium. Nrxn1a-*α* also appeared to have a notably higher signal than Nrxn1a-*β*, which correlates with the larger H3K4me3 peak and known higher expression of the α isoform in mammals (Anderson et al., 2015). Thus, *in situ* expression patterns could be consistent with a scenario by which Nrxn1a*-α* and -*β* isoforms have both overlapping and unique neurodevelopmental functions that might result in behavioral diversity. Due to the small size of the Nrxn1a-γ isoform-specific exon (33 bp), we were unable to perform comparable *in situ* analyses.

**Fig. 2.**
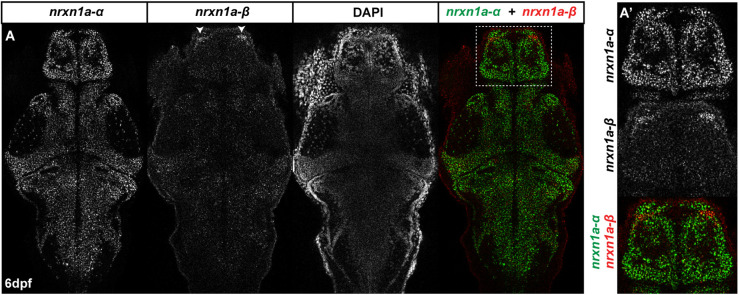
***nrxn1a-α* and *-β* transcripts have distinct brain expression patterns.** (A,A′) *In situ* hybridization chain reaction (HCR) images of *nrxn1a* isoform-specific exons in wild-type larval brain at 6 dpf. Expression of *nrxn1a-α* and -*β* RNA is largely overlapping, although *nrxn1a-β* shows enrichment in the pallium (arrowheads). The boxed area indicates the telencephalon, which is shown enlarged in A′ to demonstrate enrichment of *nrxn1a-β* RNA in the dorsal telencephalon, the pallium. Images are representative of *n=*12 zebrafish larvae.

### Isoform-specific *nrxn1a* deletions cause distinct sensorimotor behavioral phenotypes

To define if and to which degree isoform-specific *Nrxn1* deletions correlate with specific behavioral phenotypes, we used CRISPR-Cas9 mediated genome editing to generate isoform-specific *nrxn1a* deletion zebrafish mutants. Specifically, we generated mutant zebrafish lines harboring deletions that delete nearly the entire *nrxn1a* locus – *nrxn1a* whole domain (*nrxn1a-WD*) – and *nrxn1a* deletions spanning isoform α-, β- and γ-specific exons (*nrxn1a-α*, *nrxn1a-β* and *nrxn1a-γ*) (Fig. 3A). Nrxn1 proteins contain multiple extracellular laminin G/neurexin/sex hormone-binding globulin (LNS) domains interspersed with epidermal growth factor (EGF)-like repeats and a single transmembrane (TM) domain. All the isoform-specific deletions are predicted to remove the start codon and to abolish the entire *nrxn1a* open reading frame for their respective isoform, whereas the *nrxn1a-WD* deletion is predicted to delete all but a small part of the first LNS domain. We further validated each deletion line with RT-PCR (Fig. 3B,D), which confirmed that isoform-specific deletions abolish the targeted transcript, whereas the *nrxn1a-WD* deletion eliminates transcripts of all isoforms. As such, all the deletions are predicted to cause loss of function.

**Fig. 3.**
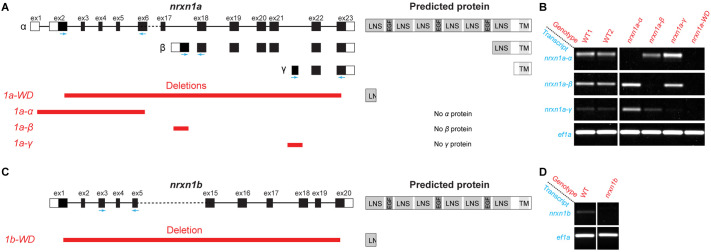
**Zebrafish *nrxn1* deletion lines.** (A) Schematic showing the exon (ex) structures of *nrxn1a* and *nrxn1b* deletion lines. *nrxn1a-WD* (*1a-WD*) deletes nearly the entire locus (only part of the first LNS remains), *nrxn1a-α*-specific line (*1a-α*) deletes *α*-specific exons, *nrxn1a-β*-specific line (*1a-β*) deletes the lone *β*-specific exon and the *nrxn1a-γ*-specific line (*1a-γ*) deletes the single *γ*-specific exon. *nrxn1a-WD* is predicted to lead to a severely truncated protein with disruption of all major domains. *nrxn1a-α*, *-*β and *-*γ lines are predicted not to yield a protein product for their respective isoforms. Blue arrows denote primer locations as indicated for RT-PCR shown in B. LNS, laminin G/neurexin/sex hormone-binding globulin domain; EGF, epidermal growth factor-like repeats; TM, transmembrane domain. (B) RT-PCR of *nrxn1a* isoform transcripts in wild-type, isoform-specific deletion mutant and whole-gene deletion mutant larval pools. (C) Schematic showing the exon (ex) structures of *nrxn1b* and the *nrxn1b* deletion line (*1b-WD*). *nrxn1b-WD* deletes nearly the entire locus (only part of the first LNS remains) and is predicted to lead to a severely truncated protein with disruption of all major domains. Blue arrows denote primer locations for RT-PCR in D. (D) RT-PCR for *nrxn1b* in wild-type and whole-gene deletion mutant larval pools.

For each mutant line, we then in-crossed heterozygous adults and analyzed behavior of wild-type, heterozygous and homozygous offspring at 6 dpf. Because *nrxn1a* crispants displayed reduced locomotion during the light-induced VMR and DF assays as well as ASR hypersensitivity, we focused subsequent behavioral analyses on these behaviors. We did not observe any differences between wild-type and heterozygous animals (Fig. S2) in any lines assayed. Therefore, for our analysis, we compared homozygous mutants (*nrxn1a^−/−^*) with their combined sibling group of homozygous wild-type (*nrxn1a^+/+^*) and heterozygous (*nrxn1a^+/−^*) animals. Compared to this sibling group, *nrxn1a-WD* and *nrxn1a-α* mutants displayed reduced movement (defined by number of bouts) during the VMR assay, which was most pronounced during the light phase of the assay, i.e. the first 8 min (Fig. 4A,B; S4A,B), thereby recapitulating the *nrxn1a* crispant results. We noticed that, compared to *nrxn1a-WD,* the phenotype was more pronounced in *nrxn1a-α* mutants, which displayed a substantial reduction in the number of bouts averaged across the entire light period (Fig. S3A,B). In contrast, both *nrxn1a-β* and *nrxn1a-γ* mutants displayed increased movement during the light phase of the VMR (Fig. 4C,D; S4C,D). This phenotype was most pronounced in *nrxn1a-β* mutants, which displayed a substantial increase in the number of bouts when averaging across the entire light period (Fig. S3C,D). This could explain why the phenotype of the *nrxn1a-WD* deletion, which also deletes Nrxn1a β and γ isoforms, is less severe than the *nrxn1a-α* deletion phenotype. In the DF assay, *nrxn1a-WD* and *nrxn1a-α* mutants displayed reduced movement, defined by average distance travelled following a flash of darkness, compared to siblings (Fig. 4F,G; S4F,G). In contrast, compared to siblings following a flash of darkness, *nrxn1a-β* mutants displayed increased movement, i.e. a greater distance travelled (Fig. 4H; S4H), while we failed to detect differences in *nrxn1a-γ* mutants (Fig. 4I; S4I). Compared to siblings, *nrxn1a-WD* mutants displayed a substantial increase in sensitivity to startling stimuli in the ASR assay (Fig. 4K; S4K). No differences in ASR were observed in *nrxn1a-α*, *nrxn1a-β* or *nrxn1a-γ* mutants (Fig. 4L-N; S4L-N), suggesting that – while *nrxn1a* is crucial to regulate ASR stimulus sensitivity – individual isoforms may functionally compensate for each other. Together, our results indicate that *in vivo* isoform-specific *nrxn1a* deletions caused distinct, and at times opposing, behavioral phenotypes. Further, we found that while the *nrxn1a-α* deletion mimics the *nrxn1a-WD* VMR and DF phenotypes, the *nrxn1a-α* deletion fails to recapitulate the *nrxn1a-WD* ASR phenotype.

**Fig. 4.**
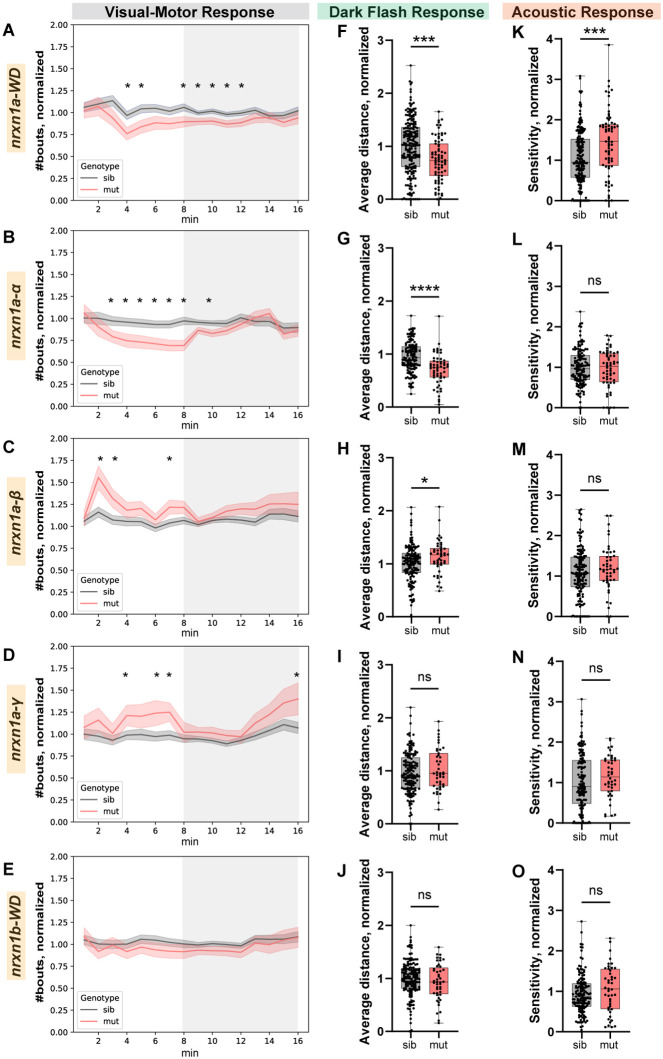
**Isoform-specific *nrxn1a* deletions cause distinct sensorimotor behavioral phenotypes.** (A-E) Plotted are the number of bouts (#bouts) moved for homozygous mutant (mut) and sibling (sib) during each minute of the VMR assay, normalized to the wild-type average for each deletion line. Plots show results of the light (left) and dark (right) phase of VMR assays, i.e. the first 8 min and last 8 min (white and gray shading, respectively). *P<0.05 (Student's *t*-test). Shaded areas (gray, red) indicate the mean±s.e.m. (F-J) Plotted is the average distance travelled following a flash of darkness for each deletion line, normalized to that of wild type. (K-O) Plotted is the sensitivity to acoustic stimuli, for each deletion line, normalized to that of wild type. *nrxn1a-WD*: *n=*75 mut, 197 sib (131 het, 66 wt)*. nrxn1a-α*: *n=*56 mut, 130 sib (79 het, 51 wt); *nrxn1a-β*: *n=*47 mut, 149 sib (104 het, 45 wt). *nrxn1a-γ*: *n=*44 mut, 154 sib (96 het, 58 wt); *nrxn1b-WD*: *n=*41 mut, 143 sib (99 het, 44 wt). Data presented are cumulative across four to five biological replicates. ns, not significant. *P<0.05, ***P<0.001, ****P<0.0001 based on Student's *t*-test. Wild-type versus heterozygous genotype comparisons are shown in Fig S2; Representative raw (non-normalized) data from individual biological replicates for each deletion line are shown in Fig S4.

### Design of an observer-independent, hands-free assay to analyze locomotion and social preferences

Given the strong association of NRXN1 with human neurodevelopmental disorders, such as autism, we next assayed social behaviors. For this, we developed an observer-independent, hands-free assay for the analysis of 5 weeks post fertilization (wpf) juvenile fish that enables simultaneous imaging of baseline locomotion and social preference (Fig. 5A-C). Briefly, we designed an arena composed of a long individual lane flanked on each end by a smaller compartment into which social stimuli, such as other fish or objects, can be placed. Unlike most arenas reported previously (Geng and Peterson, 2019; Ogi et al., 2021; Geng et al., 2022; Dreosti et al., 2015), the testing lanes and end compartments are visually separated in our design by micro-controlled polymer dispersed liquid crystal (PDLC) films (MEGICOLIM; Hefei Shiwei Technology Co., Ltd., Hefei, Anhui, China) (Fig. 5C). PDLC films consist of liquid crystal droplets suspended in a polymer matrix, which scatter light and appear opaque by default but align and become transparent when voltage is applied. Here, an Arduino Nano microcontroller (Arduino, Monza, Italy; product code A000005) applies the voltage to control opacity, thereby eliminating the need for mechanical or moving parts that might interfere with to-be-observed behavior. To increase experimental throughput, we constructed a multiplex arena consisting of 14 individually controllable arenas at a 7×2 side-by-side configuration, allowing us to robustly test social interaction activity of 14 juvenile zebrafish at 5 wpf (Fig. 5A-C). For all experiments, we placed a ‘tester’ fish in the larger compartment of the arena, an age-matched ‘stimulus’ fish in one of the small compartments and a novel object (black acrylic rectangular prism) in the other small compartment. Following placement of a tester fish into the testing arena and its acclimation, movement and location of the fish were recorded for 10 min – a period defined as ‘baseline period’ – with the PDLC windows switched to opaque. Then, the PDLC windows were switched to transparent (‘windows open’ in Fig. 5), and the responses of the fish to social and novel object stimuli were recorded for an additional 10 min (Fig. 5D). During this ‘social period’, social preference was calculated for each fish by subtracting the time spent in the social zone during the baseline period from the time spent in the social zone during the social period, thereby providing a ‘social preference’ metric between −1 and +1, with higher positive values indicating more time spent in the social zone during the social period than during the baseline period.

**Fig. 5.**
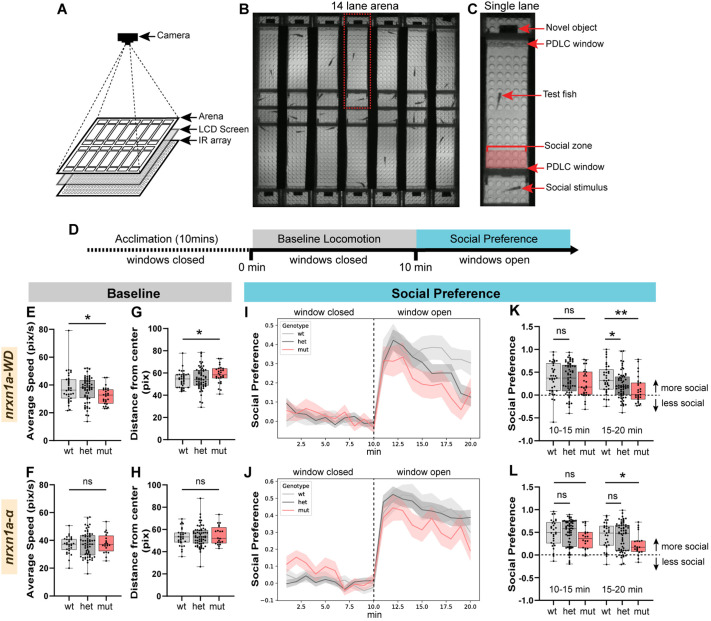
***nrxn1a* deletions lead to reduced social preference in an observer-independent, hands-free behavioral assay.** (A) Schematic of the juvenile zebrafish behavior apparatus. The arena is illuminated from below by an IR array and an LDC screen. Camera recording is from above. (B,C) Still image of a video recording of the 14-lane arena when in use (B). One lane (boxed area) is shown enlarged in C, with the test fish indicated in the main testing lane flanked by two smaller end compartments; also indicated are the social stimulus fish and novel object. The testing lane is separated by a PDLC window, the social zone (indicated in red) is defined as the area immediately adjacent to the social stimulus. (D) Schematic of the testing paradigm. Acclimation: zebrafish were allowed to acclimate to the apparatus with PDLC windows closed for 10 min. Baseline locomotion: zebrafish in the apparatus with PDLC windows closed were recorded for 10 min. Social Preference: zebrafish in the apparatus with PDLC windows open were assayed for an additional 10 min. (E-H) Average speed in pixels per second (pix/s) (E,F) and average distance from the center of the well in pixels (pix) (G,H) during the baseline period for *nrxn1a-WD* (E) and *nrxn1a-α* deletion mutants compared to siblings. (I-J) Social Preference over time (20 min) for *nrxn1a-WD* (I) and *nrxn1a-α* (J) deletion mutants (mut) compared to wild-type (wt) and heterozygous (het) siblings. Social preference is calculated for each fish by subtracting the time spent in the social zone during baseline period from the time spent in the social zone during the social period, thereby providing a metric between −1 and +1 with positive values indicating more time spent in the social zone during the social period than during the baseline period. Note the increase in social preference when the window opens after 10 min (dashed vertical line). Shaded areas (light gray, dark gray, red) indicate the mean±s.e.m. (K,L) Social Preference during the first (10-15 min) and second (15-20 min) half of the social preference assay for *nrxn1a-WD* (K) and *nrxn1a-α* (L) deletion mutants (mut) compared to wild-type (wt) and heterozygous (het) siblings. ns, not significant; *P<0.05, **P<0.01 based on Student's *t*-test or one-way ANOVA. Box plots show median (line) and interquartile range (box, 25th–75th percentile), with whiskers extending to the minimum and maximum values; individual data points represent single fish.

### *nrxn1a* deletions cause social-interaction deficits

By using this system, we were able to assess both baseline and social behavior in *nrxn1a-WD* and *nrxn1a-α* deletion lines. Importantly, at 5 wpf, homozygous and heterozygous *nrxn1a* animals appeared indistinguishable regarding total body length from age-matched wild-type siblings (Fig. S5A). To assess if *nrxn1a* function is required for behavior during the baseline period, we measured average speed as well as center-avoidance (measured as distance of the fish from the center of the long-arm compartment). During the baseline period, neither *nrxn1a-WD* nor *nrxn1a-α* heterozygotes displayed differences compared with wild-type animals (Fig. 5E-H). Compared to wild-type siblings, *nrxn1a-WD* mutants displayed reduced speed (Fig. 5E) and an increase in their distance from the center compared (Fig. 5G). In contrast, we did not observe differences in *nrxn1a-α* mutants for either of these metrics (Fig. 5F,H), suggesting that, for baseline parameters, other *nrxn1a* isoforms can compensate for *nrxn1a-α* loss. During the social preference period, wild-type sibling animals displayed a robust social preference that slightly decayed over the 10-min period (Fig. 5I-L). While homozygous *nrxn1a-WD* mutants exhibited a similar initial social preference (Fig. 5I,K), it decreased substantially more in the second half of the assay compared with wild type (Fig. 5K). Interestingly, heterozygous *nrxn1a-WD* mutants exhibited an intermediate phenotype that was also substantially different from wild-type fish in the second half of the assay (Fig. 5K). In contrast, wild type and heterozygous *nrxn1a-α* mutants displayed similar social preferences (Fig. 5J,L); and, although *nrxn1a-α* homozygous mutant animals displayed a substantially reduced social preference compared to siblings (Fig. 5L), this reduction was less severe than for *nrxn1a-WD* mutants. Together, these data define a clear role for *nrxn1a-α* in social preference but suggest that larger deletions – which also encompass the β and γ isoforms – cause more severe phenotypes. Therefore, our results from both larval and juvenile animals indicate that deletions that differentially affect Nrxn1 isoforms can have varied effects on behavior, emphasizing the importance of considering the type of deletion when interpreting clinical alleles.

## DISCUSSION

In this study, we demonstrated that deletions of zebrafish Nrxn1 isoforms cause both unique and partially overlapping behavioral phenotypes. By generating matched *nrxn1a* isoform-specific and whole locus deletions, and measuring quantifiable behavioral features using previously validated assays, we showed that deletions of isoform-specific exon groups shape discrete behavioral domains, while the full-locus deletion produces combined or more severe phenotypes. These findings provide the first direct comparison of isoform-selective Nrxn1 loss *in vivo* and underscore the importance of understanding the specific genomic architecture of NRXN1 deletions when considering pathophysiology, particularly, given the substantial inter-individual variability in deletion boundaries.

While mammalian α*,* β and γ isoforms of NRXN1 have been previously described, their individual behavioral functions have been incompletely explored. Indeed, most of the literature to date regarding NRXN1 is focusing on the NRXN1-α isoform, which has been shown to have important roles in social behavior, locomotion and anxiety (Grayton et al., 2013; Armstrong et al., 2020; Xu et al., 2023). However, there are few reports of specific roles for isoforms NRXN1-β and -γ. Recent work in *C. elegans*, which only has α and γ isoforms, has shown a specific role for nrx1-γ in a behavioral response to food deprivation (Bastien et al., 2023), highlighting the need for further studies that address isoform-specific functions. Our study is the first to consider all Nrxn1 isoforms within the same experimental paradigm, which allows comparison across mutant lines. Similar to prior studies in mice, our findings support a role for NRXN1 in social behavior, locomotion and thigmotaxis – i.e. the behavioral response to tactile stimuli (as measured by distance from the center) – that has been previously suggested as a measure of anxiety in zebrafish (Champagne et al., 2010). Combined, our results support a model in which α, β and γ isoforms contribute in both shared and distinct ways, which is discussed more thoroughly in the following paragraph. In addition, for each of the *nrxn1a* mutant lines, our data showed that larval behavior, which roughly corresponds to the pre-natal stage in humans (Weinschutz Mendes et al., 2023), is dysfunctional prior to the emergence of more-complex behavioral deficits, supporting a neurodevelopmental origin of *NRXN1* pathogenesis. This contrasts with a recent report indicating that zebrafish lacking both *nrxn1a* and *nrxn1b* do not exhibit detectable behavioral phenotypes at the larval stage (Nguyen et al., 2025 preprint). Differences in experimental design, including the temporal resolution of behavioral assays and the use of sibling versus non-sibling controls, may contribute to these differing observations. Notably, our analyses leverage high-temporal resolution behavioral measurements and within-clutch sibling controls, which may increase sensitivity to early developmental phenotypes.

Our larval phenotyping revealed that *nrxn1a-α* and *nrxn1a-β* mutants have opposing behavioral phenotypes in the VMR and DF assays. Additionally, we found that *nrxn1a-WD* mutants lacking both the *nrxn1a-α* and *nrxn1a-β* have intermediate VMR and DF phenotypes that are less severe than *nrxn1a-α*-only mutants. Together, these data support a model whereby transcripts arising from distinct promoters may drive opposing effects. However, we cannot rule out that isoform-specific deletions may cause changes in splicing or yield compensatory expression changes of other isoforms, which could underlie the observed opposing phenotypes. In contrast to the VMR and DF phenotypes, for the ASR, we only observe a hypersensitivity phenotype for the *nrxn1a-WD* deletion. This result suggests that, in contrast to the VMR and DF response, Nrxn1a-α, -β and -γ isoforms may function redundantly to regulate the ASR, such that a phenotype emerges only when all isoforms are deleted. Similarly, in juvenile phenotyping, *nrxn1a-WD* mutants display more-severe social phenotypes and additional phenotypes that are not present in *nrxn1a-α* mutants, further supporting a model in which Nrxn1a isoforms function redundantly to support juvenile behavior. While the mechanisms underlying the behavioral differences between isoform-specific deletions and whole-gene deletions remain unclear, they may reflect isoform-specific functions and/or compensatory processes that differ between partial and complete loss-of-function. Overall, our data support a complex picture of Nrxn1a isoform function, whereby isoforms perform both unique and shared functions that are both behaviorally and temporally specific.

The complexity of our results mirrors the complexity of clinical *NRXN1* deletions. Since *NRXN1* deletions are non-recurrent, non-related individuals have unique deletions. As such, these deletions can have specific effects on NRXN1 function. Previous studies have suggested NRXN1-α loss of function caused by deletions within the 5′ region of *NRXN1* as a main mechanism underlying the association of NRXN1 with neurodevelopmental phenotypes (Lowther et al., 2017). However, recent results have demonstrated that 5′ and 3′ NRXN1 deletions cause distinct functional outcomes via different mechanisms (Flaherty et al., 2019; Fernando et al., 2025). Our data support this view and provide an additional layer of complexity, suggesting that the degree to which specific deletions affect specific NRXN1 isoforms (α*,* β*,* γ) should be considered.

There are numerous reports of zebrafish social preference assays in the literature, most of which use adult animals assayed one at a time, and which usually require physical intervention to either provide or reveal a social cue (Geng and Peterson, 2019; Ogi et al., 2021). To solve the need for physical intervention, one group has previously employed the use of controllable electrochromic film, a material that changes its optical transparency in response to an applied electrical voltage, with good effect (Stednitz et al., 2018). More recently, moderate-throughput approaches, focusing on juvenile stage animals have been developed (Geng et al., 2022; Dreosti et al., 2015). However, previous moderate-throughput behavioral arenas to study social interaction in zebrafish have required to either net social stimulus fish into the arena (Dreosti et al., 2015) or to physically remove an opaque separator (Geng et al., 2022) for social presentation. To reduce the need for physical interventions that have high potential to interfere with behaviors of interest and to allow for precise control of stimulus presentation, we used Arduino Nano-microcontrolled controlled PDLC films. Compared to electrochromic film, PDLC film is less expensive, responds more quickly and provides improved transparency. It is important to note that, while these types of preference assay are widely used to assess social behavior in the field, the readout may also be influenced by non-social factors, including alterations in motor function or visual perception. Nonetheless, by using this approach, we were able to quantify social preference of wild-type animals and observe social phenotypes in animals lacking *nrxn1a*. By combining PDLC film and a multiplexed setup, this novel platform provides a moderate-throughput system with external temporal control over stimuli presentation, which represents a significant advance in the field.

## MATERIALS AND METHODS

### Experimental model details

Experiments were conducted using 6 days post fertilization (dpf) larval zebrafish and 5 weeks post fertilization (wpf) juvenile zebrafish with a body length of ∼10-15 mm (see Fig. S5) (*Danio rerio*, TLF strain). Larvae were raised in E3 medium at 29°C on a 14:10 h light cycle, and juvenile and breeding adult zebrafish were maintained at 28°C on a 14:10 h light cycle. All animal protocols were approved by the University of Pennsylvania Institutional Animal Care and Use Committee (IACUC).

### Crispant experiments

Crispants were generated as described previously (Kroll et al., 2021). Briefly, three guide RNAs (gRNAs) targeting three different regions across the *nrxn1a* and *nrxn1b* loci were designed using ChopChop v.3 (https://chopchop.cbu.uib.no/) (Table S1). Custom Alt-R CRISPR-Cas9 crRNAs (IDT) were annealed with Alt-R™ CRISPR-Cas9 tracrRNA [#1072533; Integrated DNA Technologies, Inc. (IDT), Coralville, IA USA] to form gRNAs that were subsequently complexed with Cas9 protein (IDT, #1081061) to make the final ribonucleoprotein (RNP) complex. Three non-targeting CRISPR RNAs (crRNAs) (#1072544, #1072545, #1072546; IDT) were used to make the RNP for controls. Single-cell wild-type (TLF) zebrafish embryos were then microinjected within 15 min of fertilization with 1 nl of RNP mix containing 357 pg (10.1 fmol) of each gRNA and 5029 pg (30.5 fmol) of Cas9. Embryos displaying acute toxicity or damage from microinjection were removed from analysis. Behavior of crispants was then assayed at 6 dpf as described below.

### Generation of stable deletion lines and genotyping

Mutant alleles were generated using CRISPR-Cas9 mutagenesis. Two gRNAs flanking the desired region, along with a third nested gRNA within the region to be deleted, were designed using ChopChop v.2 (https://chopchop.cbu.uib.no/). RNP complexes were then made and injected into single-cell wild-type (TLF) zebrafish embryos, as described above under ‘Crispant experiments’. F_0_-injected larvae were raised and outcrossed to identify and establish heterozygous carrier lines. Mutant lines were identified and subsequently genotyped by PCR, using primers flanking the outermost gRNA target sites. Allele sequences were obtained by Sanger-sequencing of resulting PCR products. For each allele, genotyping was performed using the two primers flanking the deletion to amplify the mutant allele together with a third nested primer to amplify the wild-type allele (Table S1). Heterozygous deletion carriers were in-crossed to generate wild-type, heterozygote and homozygote animals for larval and juvenile behavioral experiments. All behavioral experiments were performed on F_3_-generation animals or later.

### RT-PCR validation of deletion lines

Heterozygous carriers of each deletion line were in-crossed and, at 6 dpf, larval tails were clipped for genotyping and heads were immediately preserved in RNAlater solution (#AM7020, Invitrogen). Heads from larvae of the same genotype were pooled (*n=*6-14), and total RNA was extracted using TRIzol (#15596026, Invitrogen). cDNA was synthesized from 100-500 ng of total RNA using SuperScript III First-Strand Synthesis System (#18080051, Invitrogen). RT-PCR was then performed using isoform-specific primers (Table S1). Two replicates were performed for each genotype.

### Hybridization chain reaction fluorescent *in situ* hybridization

Endogenous *nrxn1a-α* and *nrxn1a-β* transcripts were detected by using hybridization chain reaction (HCR) fluorescent *in situ* hybridization (Molecular Instruments, Los Angeles, CA, USA). Probes for *nrxn1a-α* were designed against the α-specific exons 1-4. Probes for *nrxn1a-β* were designed against exon 1 of the β transcript. HCR buffers, probes and hairpins were purchased from Molecular Instruments. Wild-type TLF larvae at 6 dpf were fixed with 4% paraformaldehyde in Dulbecco's phosphate-buffered saline solution overnight at 4°C and staining was performed as previously described (Shainer et al., 2023).

### Larval behavioral experiment and analysis

Crispants and stable mutant lines were assayed for sensorimotor phenotypes as previously described (Campbell et al., 2023; Maroni et al., 2024 preprint). Briefly, larvae were arrayed in a 100-well plate and assayed for the VMR, light flash (LF) response, dark flash (DF) response, and acoustic startle response (ASR). Recorded videos were then tracked and analyzed offline using previously published Python codes (Campbell et al., 2023).

### Juvenile behavior arena

The testing arena consists of a 2×7 array of testing lanes and is made of laser-cut acrylic. Each testing lane is 80 mm×30 mm (holds ∼18 ml of water) and is flanked on each end by a compartment of 18 mm×30 mm (holds ∼5 ml of water). The bottom of the arena is clear transparent acrylic which allows fish to observe an LCD screen that is placed underneath the arena and backlit by LED white lights. An IR array also illuminates from below which is detected by a Chameleon3 monochrome camera mounted above (CM3-U3-13Y3M-CS; Teledyne FLIR, Wilsonville, OR, USA) and fitted with an M30.5 LP800 Near-IR longpass filter (Midwest Optical Systems, Palatine, IL, USA) at 20 fps. Side walls of the arena are opaque white acrylic to prevent fish in adjacent lanes from visualizing one another. The end walls of the lanes are fitted with polymer dispersed liquid crystal (PDLC) windows (MEGICOLIM; Hefei Shiwei Technology Co., Ltd., Hefei, Anhui, China) which are opaque but become transparent when triggered by an Arduino Nano microcontroller (Arduino, Monza, Italy; product code A000005), allowing fish to observe what is placed in the end compartments (i.e. social stimulus fish or novel object). The entire testing arena is contained in a blackout-curtain enclosure to minimize external interference. Information regarding hardware and the code for the juvenile behavioral arena is openly accessible at https://github.com/pdcampbell/ZF-juvenile-rig.

### Juvenile behavior experiment and analysis

Heterozygous deletion carriers were in-crossed and progeny were raised to 5 wpf alongside age-matched wild-type fish (TLF strain) to serve as social stimuli. At 5 wpf fish were moved from their housing tanks and placed into either the testing lanes (deletion fish to be tested) or the end compartments (wild-type social stimuli). Fish were allowed to acclimate to the environment for 10 min while white light was projected from below by the LCD screen. Then, baseline movements were recorded for 10 min. PDLC films were then triggered to open and social preference was recorded for an additional 10 min. Tested fish were then individually removed from the testing arena and genotyped. Offline tracking of recorded videos was performed with custom-written Python codes. To identify objects in each well, each image of the video was background subtracted, Gaussian blurred and thresholded. Fish that were improperly tracked (i.e. no objects were detected in the well or >1 object was detected) in >10% of the frames or that had >200 consecutive improperly tracked frames were discarded (∼5% of fish). Remaining fish displayed a high tracking accuracy (>99% of frames tracked correctly, *n=*254 fish). Missing frames were filled in using the SciPy.interpolate function (https://docs.scipy.org/doc/scipy/reference/interpolate.html). Fish that did not move for >1000 consecutive frames (i.e. 50 s) during the baseline period were also excluded from analysis as they were deemed to be exhibiting freezing behavior. Excluded fish spanned all genotypes and were not clustered in one group. Object centroids for remaining fish were then used to calculate average speed, position in the well relative to the social stimulus and position in the well relative to the center. Average speed was computed as the distance travelled by the centroid over the time spent moving. Social preference was calculated for each fish by subtracting the fraction of time in the area nearest the social stimulus (social zone) during the last 5 min of the baseline period when the PDLC was closed from the fraction of time in the social zone when the PDLC was open. Distance from the center was computed as the distance between the centroid of the fish and the center of the well. Data were binned into 1 min bins with an average computed for each 1 min bin. Length of fish was measured from the videos in ImageJ. Information regarding tracking and analysis code for the juvenile behavioral experiments is openly accessible at https://github.com/pdcampbell/ZF-juvenile-rig.

## Supplementary Material

10.1242/dmm.052862_sup1 Supplementary information

Table S1.

## References

[DMM052862C1] Anderson, G. R., Aoto, J., Tabuchi, K., Földy, C., Covy, J., Yee, A. X., Wu, D., Lee, S.-J., Chen, L., Malenka, R. C. et al. (2015). β-neurexins control neural circuits by regulating synaptic endocannabinoid signaling. *Cell* 162, 593-606. 10.1016/j.cell.2015.06.056 26213384 PMC4709013

[DMM052862C2] Armstrong, E. C., Caruso, A., Servadio, M., Andreae, L. C., Trezza, V., Scattoni, M. L. and Fernandes, C. (2020). Assessing the developmental trajectory of mouse models of neurodevelopmental disorders: social and communication deficits in mice with Neurexin 1α deletion. *Genes Brain Behav.* 19, e12630. 10.1111/gbb.12630 31823470

[DMM052862C3] Bastien, B. L., Cowen, M. H. and Hart, M. P. (2023). Distinct neurexin isoforms cooperate to initiate and maintain foraging activity. *Transl. Psychiatry* 13, 367. 10.1038/s41398-023-02668-z 38036526 PMC10689797

[DMM052862C4] Bogdanović, O., Smits, A. H., de la Calle Mustienes, E., Tena, J. J., Ford, E., Williams, R., Senanayake, U., Schultz, M. D., Hontelez, S., van Kruijsbergen, I. et al. (2016). Active DNA demethylation at enhancers during the vertebrate phylotypic period. *Nat. Genet.* 48, 417-426. 10.1038/ng.3522 26928226 PMC5912259

[DMM052862C5] Burgess, H. A. and Granato, M. (2007a). Sensorimotor gating in larval zebrafish. *J. Neurosci.* 27, 4984-4994. 10.1523/JNEUROSCI.0615-07.2007 17475807 PMC6672105

[DMM052862C6] Burgess, H. A. and Granato, M. (2007b). Modulation of locomotor activity in larval zebrafish during light adaptation. *J. Exp. Biol.* 210, 2526-2539. 10.1242/jeb.003939 17601957

[DMM052862C7] Campbell, P. D., Lee, I., Thyme, S. and Granato, M. (2023). Mitochondrial proteins encoded by the 22q11.2 neurodevelopmental locus regulate neural stem and progenitor cell proliferation. *Mol. Psychiatry* 28, 3769-3781. 10.1038/s41380-023-02272-z 37794116 PMC10730408

[DMM052862C8] Castronovo, P., Baccarin, M., Ricciardello, A., Picinelli, C., Tomaiuolo, P., Cucinotta, F., Frittoli, M., Lintas, C., Sacco, R. and Persico, A. M. (2020). Phenotypic spectrum of NRXN1 mono- and bi-allelic deficiency: a systematic review. *Clin. Genet.* 97, 125-137. 10.1111/cge.13537 30873608

[DMM052862C9] Champagne, D. L., Hoefnagels, C. C. M., de Kloet, R. E. and Richardson, M. K. (2010). Translating rodent behavioral repertoire to zebrafish (Danio rerio): relevance for stress research. *Behav. Brain Res.* 214, 332-342. 10.1016/j.bbr.2010.06.001 20540966

[DMM052862C10] Dachtler, J., Ivorra, J. L., Rowland, T. E., Lever, C., Rodgers, R. J. and Clapcote, S. J. (2015). Heterozygous deletion of α-neurexin I or α-neurexin II results in behaviors relevant to autism and schizophrenia. *Behav. Neurosci.* 129, 765-776. 10.1037/bne0000108

[DMM052862C11] Dreosti, E., Lopes, G., Kampff, A. R. and Wilson, S. W. (2015). Development of social behavior in young zebrafish. *Front. Neural. Circuits* 9, 39. 10.3389/fncir.2015.00039 26347614 PMC4539524

[DMM052862C12] Emran, F., Rihel, J. and Dowling, J. E. (2008). A behavioral assay to measure responsiveness of zebrafish to changes in light intensities. *J. Vis. Exp.* 20, 923. 10.3791/923 PMC2879884 19078942

[DMM052862C13] Fernando, M. B., Fan, Y., Zhang, Y., Tokolyi, A., Murphy, A. N., Kammourh, S., Deans, P. J. M., Ghorbani, S., Onatzevitch, R., Pero, A. et al. (2025). Phenotypic complexities of rare heterozygous neurexin-1 deletions. *Nature* 642, 710-720. 10.1038/s41586-025-08864-9 40205044

[DMM052862C14] Flaherty, E., Zhu, S., Barretto, N., Cheng, E., Deans, P. J. M., Fernando, M. B., Schrode, N., Francoeur, N., Antoine, A., Alganem, K. et al. (2019). Neuronal impact of patient-specific aberrant NRXN1α splicing. *Nat. Genet.* 51, 1679-1690. 10.1038/s41588-019-0539-z PMC7451045 31784728

[DMM052862C15] Geng, Y. and Peterson, R. T. (2019). The zebrafish subcortical social brain as a model for studying social behavior disorders. *Dis. Model. Mech.* 12, dmm039446. 10.1242/dmm.039446 31413047 PMC6737945

[DMM052862C16] Geng, Y., Zhang, T., Alonzo, I. G., Godar, S. C., Yates, C., Pluimer, B. R., Harrison, D. L., Nath, A. K., Yeh, J.-R. J., Drummond, I. A. et al. (2022). Top2a promotes the development of social behavior via PRC2 and H3K27me3. *Sci. Adv.* 8, eabm7069. 10.1126/sciadv.abm7069 36417527 PMC9683714

[DMM052862C17] Grayton, H. M., Missler, M., Collier, D. A. and Fernandes, C. (2013). Altered social behaviours in neurexin 1α knockout mice resemble core symptoms in neurodevelopmental disorders. *PLoS ONE* 8, e67114. 10.1371/journal.pone.0067114 PMC3696036 23840597

[DMM052862C18] Harrison, P. W., Amode, M. R., Austine-Orimoloye, O., Azov, A. G., Barba, M., Barnes, I., Becker, A., Bennett, R., Berry, A., Bhai, J. et al. (2024). Ensembl 2024. *Nucleic Acids Res.* 52, D891-D899. 10.1093/nar/gkad1049 37953337 PMC10767893

[DMM052862C19] Hu, Y., Flockhart, I., Vinayagam, A., Bergwitz, C., Berger, B., Perrimon, N. and Mohr, S. E. (2011). An integrative approach to ortholog prediction for disease-focused and other functional studies. *BMC Bioinformatics* 12, 357. 10.1186/1471-2105-12-357 21880147 PMC3179972

[DMM052862C20] Kimmel, C. B., Patterson, J. and Kimmel, R. O. (1974). The development and behavioral characteristics of the startle response in the zebra fish. *Dev. Psychobiol.* 7, 47-60. 10.1002/dev.420070109 4812270

[DMM052862C21] Kroll, F., Powell, G. T., Ghosh, M., Gestri, G., Antinucci, P., Hearn, T. J., Tunbak, H., Lim, S., Dennis, H. W., Fernandez, J. M. et al. (2021). A simple and effective f0 knockout method for rapid screening of behaviour and other complex phenotypes. *eLife* 10, e59683. 10.7554/eLife.59683 33416493 PMC7793621

[DMM052862C22] Laarakker, M. C., Reinders, N. R., Bruining, H., Ophoff, R. A. and Kas, M. J. H. (2012). Sex-dependent novelty response in neurexin-1α mutant mice. *PLoS ONE* 7, e31503. 10.1371/journal.pone.0031503 PMC3278455 22348092

[DMM052862C23] Lowther, C., Speevak, M., Armour, C. M., Goh, E. S., Graham, G. E., Li, C., Zeesman, S., Nowaczyk, M. J. M., Schultz, L.-A., Morra, A. et al. (2017). Molecular characterization of NRXN1 deletions from 19,263 clinical microarray cases identifies exons important for neurodevelopmental disease expression. *Genet. Med.* 19, 53-61. 10.1038/gim.2016.54 27195815 PMC4980119

[DMM052862C24] Maroni, M. J., Barton, M., Lynch, K., Deshwar, A. R., Campbell, P. D., Millard, J., Lee, R., Cohen, A., Ahmad, R., Paranjapye, A. et al. (2026). Loss of DOT1L function disrupts neuronal transcription, animal behavior, and leads to a novel neurodevelopmental disorder. *Brain* 149, 343-359. 10.1093/brain/awaf212 40494548 PMC12782159

[DMM052862C25] Marsden, K. C., Jain, R. A., Wolman, M. A., Echeverry, F. A., Nelson, J. C., Hayer, K. E., Miltenberg, B., Pereda, A. E. and Granato, M. (2018). A Cyfip2-dependent excitatory interneuron pathway establishes the innate startle threshold. *Cell Rep.* 23, 878. 10.1016/j.celrep.2018.03.095 29669291 PMC6642828

[DMM052862C26] Nguyen, Q., Guo, F., Mowry, B., Das, J. and Giacomotto, J. (2026). Modelling mental disorders in zebrafish. Neurexins severely modulate anxiety, social behaviours and aggression. *Mol. Psychiatry*. Epub ahead of print. PMID: 42362769. doi:10.1038/s41380-026-03721-1 10.1038/s41380-026-03721-1 PMC13668736 42362769

[DMM052862C27] Ogi, A., Licitra, R., Naef, V., Marchese, M., Fronte, B., Gazzano, A. and Santorelli, F. M. (2021). Social preference tests in zebrafish: a systematic review. *Front. Vet. Sci.* 7, 590057. 10.3389/fvets.2020.590057 33553276 PMC7862119

[DMM052862C28] Shainer, I., Kuehn, E., Laurell, E., Al Kassar, M., Mokayes, N., Sherman, S., Larsch, J., Kunst, M. and Baier, H. (2023). A single-cell resolution gene expression atlas of the larval zebrafish brain. *Sci. Adv.* 9, eade9909. 10.1126/sciadv.ade9909 36812331 PMC9946346

[DMM052862C29] Stednitz, S. J., McDermott, E. M., Ncube, D., Tallafuss, A., Eisen, J. S. and Washbourne, P. (2018). Forebrain control of behaviorally driven social orienting in zebrafish. *Curr. Biol.* 28, 2445-2451.e3. 10.1016/j.cub.2018.06.016 30057306 PMC6082377

[DMM052862C30] Südhof, T. C. (2017). Synaptic neurexin complexes: a molecular code for the logic of neural circuits. *Cell* 171, 745-769. 10.1016/j.cell.2017.10.024 29100073 PMC5694349

[DMM052862C31] Weinschutz Mendes, H., Neelakantan, U., Liu, Y., Fitzpatrick, S. E., Chen, T., Wu, W., Pruitt, A., Jin, D. S., Jamadagni, P., Carlson, M. et al. (2023). High-throughput functional analysis of autism genes in zebrafish identifies convergence in dopaminergic and neuroimmune pathways. *Cell Rep.* 42, 112243. 10.1016/j.celrep.2023.112243 36933215 PMC10277173

[DMM052862C32] Wolman, M. A., Jain, R. A., Liss, L. and Granato, M. (2011). Chemical modulation of memory formation in larval zebrafish. *Proc. Natl. Acad. Sci. USA* 108, 15468-15473. 10.1073/pnas.1107156108 21876167 PMC3174630

[DMM052862C33] Xu, B., Ho, Y., Fasolino, M., Medina, J., O'Brien, W. T., Lamonica, J. M., Nugent, E., Brodkin, E. S., Fuccillo, M. V., Bucan, M. et al. (2023). Allelic contribution of Nrxn1α to autism-relevant behavioral phenotypes in mice. *PLoS Genet.* 19, e1010659. 10.1371/journal.pgen.1010659 PMC9997995 36848371

[DMM052862C34] Zweier, C., de Jong, E. K., Zweier, M., Orrico, A., Ousager, L. B., Collins, A. L., Bijlsma, E. K., Oortveld, M. A. W., Ekici, A. B., Reis, A. et al. (2009). CNTNAP2 and NRXN1 are mutated in autosomal-recessive pitt-hopkins-like mental retardation and determine the level of a common synaptic protein in Drosophila. *Am. J. Hum. Genet.* 85, 655-666. 10.1016/j.ajhg.2009.10.004 19896112 PMC2775834

