# Supplementary material for: Unique and overlapping behavioral effects of isoform-specific NRXN1 deletions
Source: Dis Model Mech. 2026 Jul 23;19(7):dmm052862. doi: 10.1242/dmm.052862 (PMC13446563; doi:10.1242/dmm.052862)
Supplement: Supplementary file 1 [file dmm-19-052862-s1.pdf]

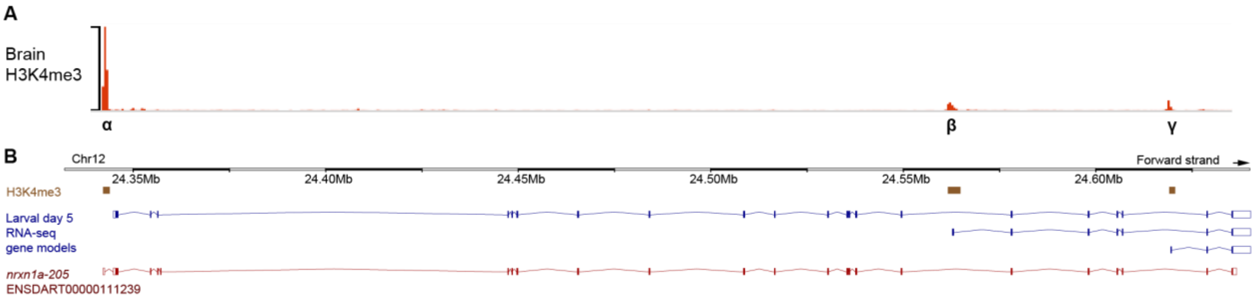

Fig. S1. *nrxn1a* has three main promoter-defined transcripts.

Transcript models from Ensembl (danRer11), supported by larval (5dpf) RNA-seq data (B), are shown relative to brain H3K4me3 enrichment from zebrafishENCODE (A).

Disease Models & Mechanisms • Supplementary information

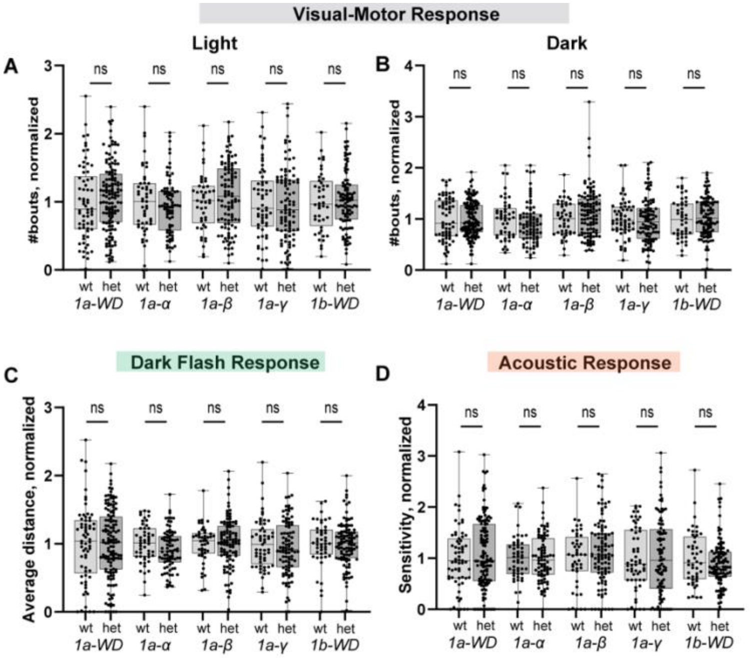

Fig. S2. (A,B) Average of normalized bouts across all light and dark minutes for each deletion line, heterozygous mutants compared to wildtypes. (C) Average distance travelled following a flash of darkness, normalized to wildtypes for each deletion line, heterozygous mutants compared to wildtypes. (D) Sensitivity to acoustic stimuli, normalized to wildtypes for each deletion line, heterozygous mutants compared to wildtypes. Numbers (n) can be found in legend of Fig 4. Statistics based on Student's t-test.

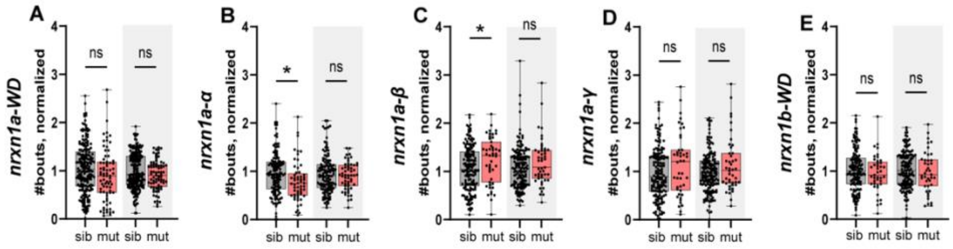

Fig. S3. (A-E) Average of normalized bouts across all light (white) and dark (gray) minutes for each deletion line. Numbers (n) can be found in legend of Fig 4. Statistics based on Student's t-test. *p<0.05 based on Student's t-test.

Disease Models & Mechanisms • Supplementary information

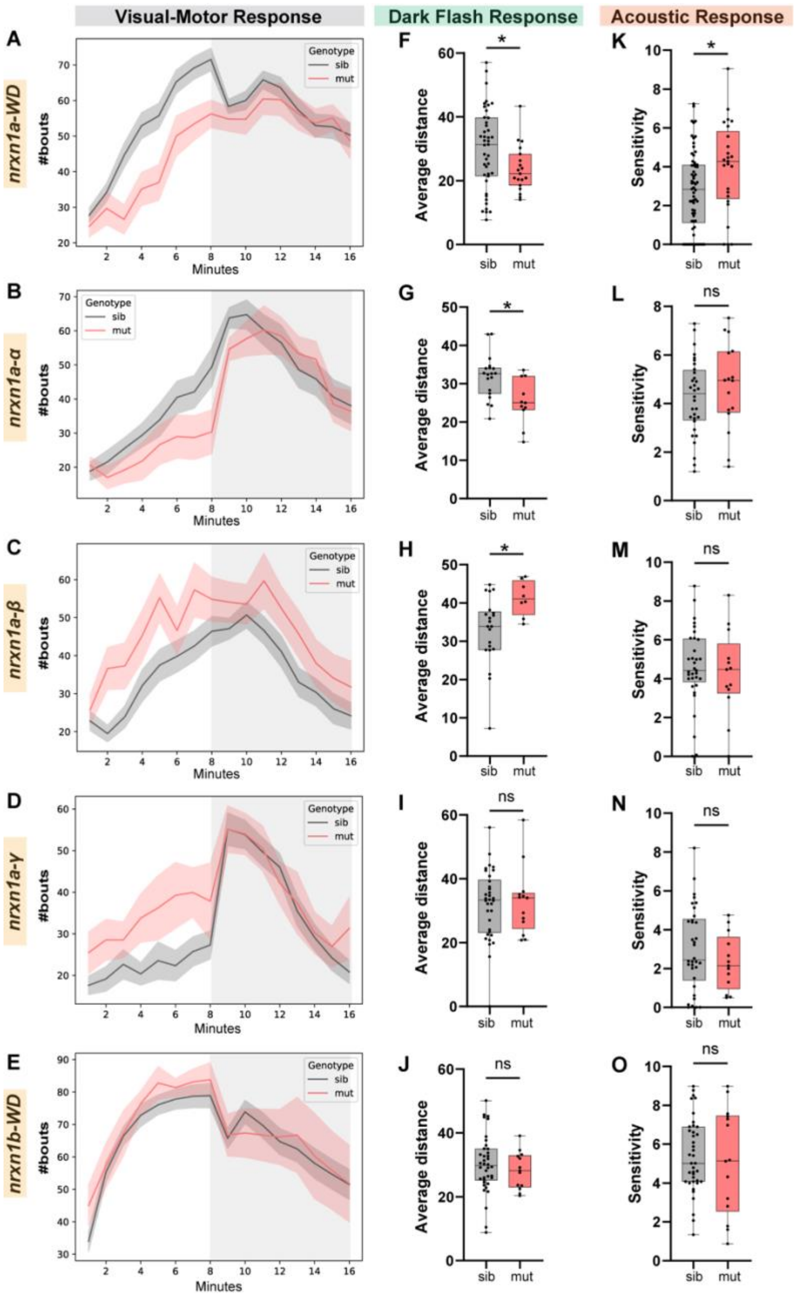

Fig. S4. Single representative replicate of raw behavioral data for each *nrxn1* deletion line. (A-E) Number of bouts moved during each minute of the VMR assay. White (first 8 minutes) and Gray (last 8 minutes) shading denote the light and dark periods of the VMR, respectively. The homozygous mutant (red) and sibling (grey) means +/- SEMs are plotted for each minute bin. (F-J) Average distance travelled following a flash of darkness. (K-O) Sensitivity to acoustic stimuli. *p<0.05 based on Student's t-test.

Disease Models & Mechanisms • Supplementary information

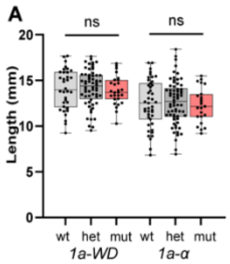

Fig. S5. Length of zebrafish at 5 weeks post-fertilization for *nrxn1a-WD* and *nrxn1a-α* deletion mutants compared to wild-type and heterozygous siblings. Stats based on One-way ANOVA.

Table S1.

Available for download at
https://journals.biologists.com/dmm/article-lookup/doi/10.1242/dmm.052862#supplementary-data

Disease Models & Mechanisms • Supplementary information
